# New host-parasite relationship: *Rocinela signata* (Aegidae: Isopoda: Crustacea) found in green sea turtles in Brazil

**DOI:** 10.1007/s11230-025-10266-4

**Published:** 2026-03-24

**Authors:** Thabata Fernanda Oliveira, Camila Miguel, Tammy Iwasa-Arai

**Affiliations:** 1 Laboratório de Diversidade Genômica, Departamento de Genética e Biologia Evolutiva, Instituto de Biociências, Universidade de São Paulo, Rua do Matão, 277, Cidade Universitária, São Paulo, SP 05508-090 Brazil; 2 Projeto Chelonia mydas – Instituto Marcos Daniel, Av. Eugênio Pachêco de Queirós, s/n, Vitória, ES 29092-170 Brazil

## Abstract

Associations between isopods and sea turtles are rarely documented. Here we report, to our knowledge, the first record of aegid isopods parasitizing sea turtles: *Rocinela signata* Schiödte & Meinert, 1879 and *Rocinela* sp. on juvenile green sea turtles, *Chelonia mydas* (Linnaeus), in the Santa Cruz Wildlife Refuge, Espírito Santo, Brazil. Between 2021 and 2023, 322 juveniles underwent morphometric and health assessments, including scoring of epibionts and ectoparasites. In total, 142 turtles (44.1%) presented fibropapillomatosis (FP). Five *R. signata* were found attached to fibropapillomas on five different turtles, and two specimens of *Rocinela* sp. were collected from two FP-free turtles—one attached to the neck and one to the eye. The consistent localization of *R. signata* on FP lesions suggests these tumors provide a favorable microhabitat, potentially via shelter and access to vascularized tissue. Given the capacity for *R. signata* to remain attached for prolonged periods, we hypothesize that *R. signata* may establish persistent parasitic associations with sea turtles and merits investigation as a potential vector of chelonid herpesvirus 5 (ChHV5). We discuss the possibility that post-disaster environmental stressors following the 2015 Fundão tailings dam collapse contributed to conditions favoring this novel host–parasite interaction.

## Introduction

Reports of cymothoidean isopods (Crustacea: Isopoda: Cymothoidea) associated with sea turtles are scarce, indicating that these relationships are uncommon. Isopods have been documented from under the peeling scutes of the green turtle, *Chelonia mydas* (Linnaeus), hawksbill turtle, *Eretmochelys imbricata* (Linnaeus), and loggerhead turtle, *Caretta caretta* (Linnaeus), in coral-reef environments (Bustard, 1976). *Eurydice* Leach, 1816 (Cirolanidae) has been observed on the eyelids of *C. mydas* in Sarawak, Malaysia (Hendrickson, 1958) and under the peeling scutes of *C. mydas*, *E. imbricata*, and *C. caretta* in coral reefs (Bustard, 1976). *Argathona macronema* (Bleeker, 1857) (Corallanidae) has been recorded on the eyes of *C. mydas* in Kenya (Monod, 1975). *Excorallana acuticauda* (Miers, 1881) (Corallanidae) has been found on the shoulders and flanks of leatherback turtles, *Dermochelys coriacea* (Vandelli), in St. Croix, U.S. Virgin Islands (Williams et al., 1996; Eckert & Eckert, 1988). *Excorallana quadricornis* (Hansen, 1890) was identified on the copulatory scars of *C. mydas* on Aves Island, Venezuela (Delaney, 1989). Additionally, *Excorallana bicornis* Lemos de Castro & Brasil Lima, 1974, *Excorallana oculata* (Hansen, 1890), and *Excorallana costata* Lemos de Castro, 1960, have been reported on the eyelids, neck, and flippers of *E. imbricata* and *C. caretta*, as well as olive ridley turtles, *Lepidochelys olivacea* (Eschscholtz), in Praia do Forte, Bahia, Brazil (Rocha Júnior et al., 2015). While some of these reported occurrences, such as those involving *Excorallana*, suggest relatively stable parasitic relationships, others likely represent accidental or temporary associations.

To our knowledge, the occurrence of isopods from the family Aegidae associating with sea turtles has not been previously recorded. Here, we report the first occurrence of *Rocinela signata* Schiödte & Meinert, 1879 (Aegidae) and *Rocinela* sp. parasitizing juvenile *C. mydas* in the Santa Cruz Wildlife Refuge, Brazil.

## Materials and methods

The Santa Cruz Wildlife Refuge (Espírito Santo, southeastern Brazil), located south of the Doce River mouth, was the study area. Between 2021 and 2023, the *Chelonia mydas* monitoring program conducted systematic captures and health assessments as part of ongoing population surveys. Monofilament nylon gillnets (stretched mesh 8 cm; length 200 m) were deployed for one week twice per year (winter and summer). Nets were set parallel to the prevailing current for 8 h soak periods and checked every 30 min.

Upon capture, each turtle underwent standard morphometric and health evaluation following Miguel et al. (2022). The presence of fibropapillomatosis (FP) was recorded as present = 1 or absent = 0. All animals were handled and released at the capture site following welfare best practices; procedures were conducted under the relevant institutional and governmental permits.

Because sea turtles commonly host epibionts (Frick & Pfaller, 2013), we quantified epibiont load (EL) on an ordinal 0–3 scale based on carapace coverage: 0 = absent, 1 = mild (≤ 30%), 2 = moderate (30–60%), and 3 = heavy (> 60%). Ectoparasite intensity (P) was similarly categorized: 0 = absent, 1 = mild (< 10 individuals), 2 = moderate (10–20), and 3 = heavy (> 20) (Miguel et al., 2022).

Isopods attached to turtles were removed, fixed and preserved in ethanol (70–100%), and stored at −20 °C. Specimens were examined to determine species composition and abundance. Identification to family and genus followed Bruce (2009), using the diagnostic keys, generic diagnoses, and morphological characters provided therein. Species-level identification was based on the descriptions of *Rocinela* by Brusca & France (1992). All material was deposited in the Crustacea Collection of the National Museum, Rio de Janeiro (MNRJ 31969–31975).

## Results

**Fig. 1:**
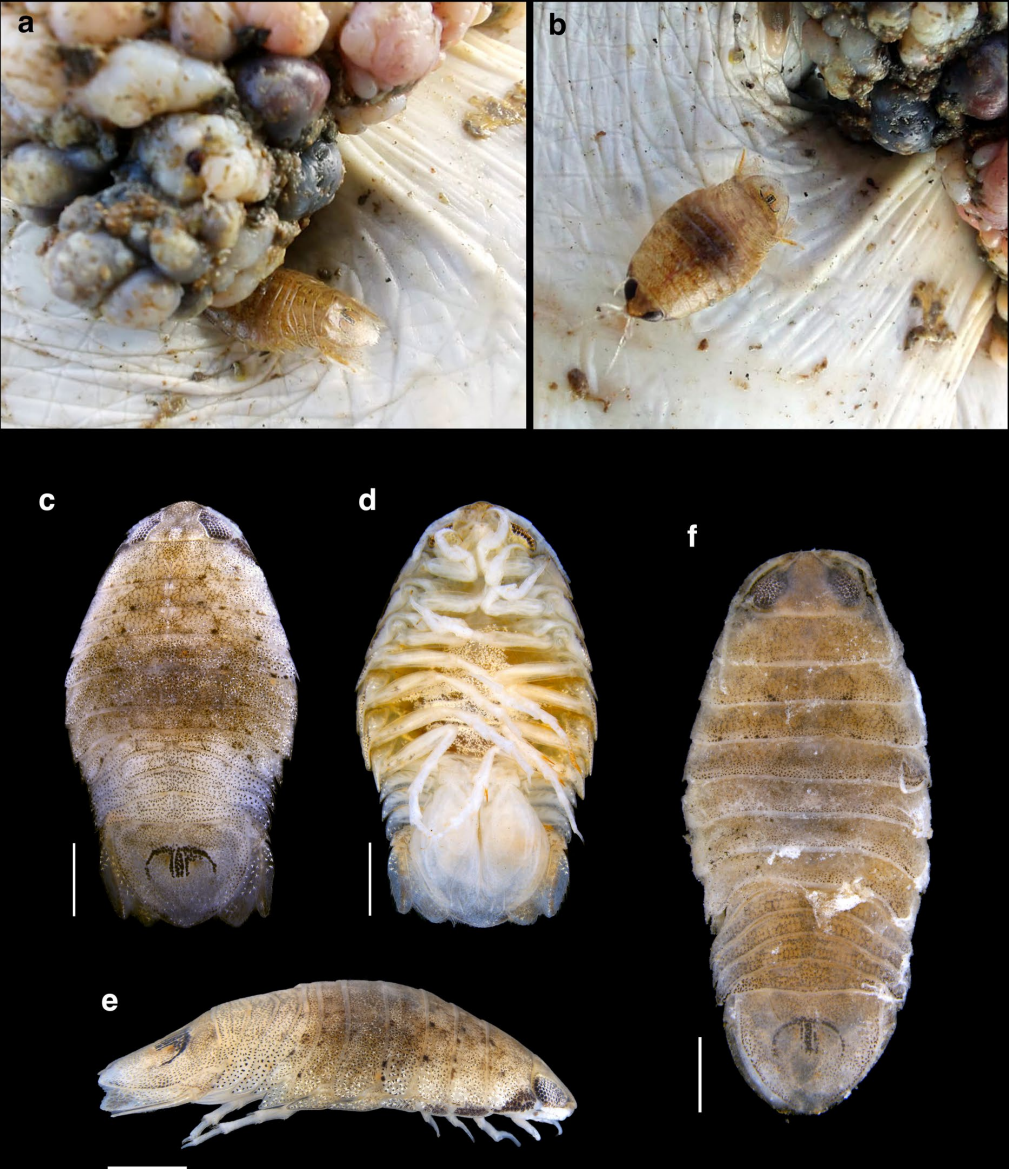
*Rocinela signata* parasitizing *Chelonia mydas*. **a** Specimen partially embedded in a fibropapilloma; **b** specimen near a fibropapilloma; **c** dorsal view; **d** ventral view; **e** lateral view; **f** *Rocinela* sp.. Scale bars = 2 mm (all panels)

A total of 322 juvenile *C. mydas* were captured and assessed; 142 (44.1%) exhibited fibropapillomatosis (FP). Five specimens of *R. signata* were found attached to fibropapillomas on five different turtles (Fig. 1). Two specimens of *Rocinela* sp. were recovered from two FP-negative *C. mydas*—one attached to the neck and the other to the eye (Fig. 1f).

The five specimens were identified as *R. signata* due to a maxillipedal palp composed of three articles; a frontal lamina thin, narrow, and arrowhead-shaped; propodi of pereopods I–III each bearing two minute spines; and the merus of pereopod I armed with three blunt spines. The rostrum is anteriorly widely rounded, and the pleotelson exhibits a distinct M-shaped pigmented region. This combination of characters is consistent with the description of *R. signata* as provided by Brusca & France (1992). A brief morphological description of both *R. signata* and *Rocinela* sp. is presented below.

### Taxonomy

- Order Isopoda Latreille, 1816
- Suborder Cymothoida Leach, 1814
- Family Aegidae White, 1850
- Genus *Rocinela* Leach, 1818
- ***Rocinela signata*** Schioedte & Meinert, 1879

Description: Body dorsally compressed. Cephalon approximately twice as wide as long; large eyes, separated by more than one eye width. Frontal lamina thin and narrow, arrowhead-shaped, narrow between the antennae, and bluntly rounded anteriorly. Antennule with a 4-articulate flagellum; antenna extending to the anterior margin of pereonite 3, with flagellum composed of 12 articles. Maxillipedal palp 3-articulate; apical article very small, bearing two stout, recurved spines; second article with two stout recurved spines, one apical seta, and one stout recurved spine on the posterior surface near the proximal margin. Pereopod I with dactylus subequal in length to propodus; propodus not expanded, bearing one small acute distal spine and one minute proximal spine; carpus with one minute spine; merus armed with three blunt spines. Pereopods II and III similar in morphology to pereopod I. Uropods extending slightly beyond the pleotelson; endopod with seven spines, exopod with six lateral spines. Pleotelson bearing a characteristic M-shaped pigmented region.

### *Rocinela* sp.

Remarks: *Rocinela* sp. closely resembles *R. signata* in overall morphology. Several diagnostic characters of *R. signata* were observed, including the general body form, the structure of the maxillipedal palp, and the proportions of the antennula and antenna, with the antennula extending to the anterior margin of pereonite 1 and the antenna extending to the anterior margin of pereonite 3 in both taxa. However, *Rocinela* sp. differs in having a rostrum that is anteriorly truncated rather than broadly rounded, and uropods that do not extend beyond the pleotelson. Although the general morphology of pereopods I–III is similar in both taxa, detailed observation of pereopodal armature was not possible due to specimen deterioration. Both specimens of *Rocinela* sp. are too damaged to allow a formal description, and additional, better-preserved material will be necessary to adequately characterise the species.

Among the seven turtles hosting isopods, body condition (BC) scores ranged from 2 (average) to 3 (good). Epibiont load (EL) was mild in six individuals and absent in one. Ectoparasite intensity (P) varied: four turtles had mild infestations, one had a moderate level, and two were heavily infested (Table 1).

**Table 1:** Characteristics of juvenile *Chelonia mydas* parasitized by *Rocinela signata* and *Rocinela* sp., including body condition (BC), epibiont load (EL), ectoparasite intensity (P), and fibropapillomatosis (FP)

Turtle ID/capture date	BC	EL	P	FP	Isopod attachment site	Isopod taxon	Deposit number
SC198/ 25 Aug 2021	3	0	1	1	Fibropapilloma	*Rocinela signata*	*MNRJ 31969*
SC210/ 29 Aug 2021	3	1	1	1	Fibropapilloma	*Rocinela signata*	*MNRJ 31970*
SC239/ 20 Jan 2022	3	1	3	1	Fibropapilloma	*Rocinela signata*	*MNRJ 31971*
SC286/ 18 Aug 2022	2	1	2	1	Fibropapilloma	*Rocinela signata*	*MNRJ 31972*
SC372/ 19 Jul 2023	2	1	3	1	Fibropapilloma	*Rocinela signata*	*MNRJ 31973*
SC381/ 20 Jul 2023	3	1	1	0	Neck	*Rocinela* sp.	*MNRJ 31974*
SC387/ 20 Jul 2023	3	1	1	0	Eye	*Rocinela* sp.	*MNRJ 31975*

The table indicates the attachment site and the isopod taxon recovered from each turtle.

*Notes:* BC = body condition (2 = average; 3 = good); EL = epibiont load (0 = absent; 1 = mild ≤30% carapace; 2 = moderate 30–60%; 3 = heavy >60%); P = ectoparasite intensity (0 = absent; 1 = <10; 2 = 10–20; 3 = >20); FP = fibropapillomatosis (0 = absent; 1 = present).

## Discussion

The discovery of *R. signata* parasitizing green turtles expands the known host range for the family Aegidae and, to our knowledge, represents the first record of an aegid isopod associating with sea turtles. The emergence of this host–parasite interaction may be linked to environmental disturbance following the Fundão tailings dam failure, which released metal-rich mine waste into regional waters (Hatje et al., 2017; Gomes et al., 2017). After this event, juvenile *C. mydas* in the Santa Cruz region exhibited health impairments consistent with heightened environmental stress and pollution (Miguel et al., 2022; Vargas et al., 2024). Although causality cannot be inferred from our data, reduced host condition could plausibly increase susceptibility to novel or opportunistic ectoparasites, facilitating an association that had not been previously documented. Turtles in poor condition also tend to carry heavier epibiont loads—barnacles, leeches, algae and other organisms—on the carapace, plastron and skin (Wyneken et al., 2006), which may further promote ectoparasite attachment and persistence.

*Rocinela signata* is widespread along the Brazilian coast and occurs both free-living and as a parasite of diverse fishes (Moreira, 1972; Moreira, 1977; Cavalcanti et al., 2013; Cardoso et al., 2017; Alves-Júnior et al., 2023). Post-disaster shifts in water quality and fish assemblages could have altered the distribution or availability of its usual fish hosts, potentially increasing encounter rates with turtles and favoring opportunistic attachment. The species has even been reported biting humans (Garzón-Ferreira, 1990; Moreira, 1977), reinforcing its behavioral plasticity and capacity to exploit atypical hosts under suitable conditions.

Members of Aegidae are typically micropredators of fishes that take a blood meal and do not remain attached for long (Bruce, 2009). By contrast, species of *Rocinela* are known to stay on the host for extended periods (Poore & Bruce, 2012). The combination of strongly hooked anterior pereopods and rapid swimming facilitates firm attachment to skin; removal often causes minor bleeding (Moreira, 1972). Such traits are consistent with the potential for more persistent parasitism on sea turtles.

Fibropapillomatosis (FP) is a benign neoplasia associated with a herpesvirus and is frequently linked to areas with intense anthropogenic stress (Aguirre & Lutz, 2004). Notably, every *R. signata* found on FP-positive turtles in our study was attached directly to fibropapillomas, suggesting that lesions provide a favorable microhabitat. Irregular tumor surfaces can shelter epibionts and ectoparasites from hydrodynamic shear, and the fibrovascular stroma offers access to vascularized tissue (Herbst, 1994). In Hawaiian green turtles with advanced FP, a variety of organisms—including mites, leeches, bacteria, yeasts, algae and trematodes—have been documented on papilloma surfaces (Aguirre et al., 1994). It is therefore reasonable to hypothesize that *R. signata* exploits the fibropapilloma microenvironment for nutrition and protection.

Given the capacity for *R. signata* to remain attached for prolonged periods and its apparent affinity for FP lesions, we propose a testable hypothesis: aegid isopods could participate in the ecology of chelonid herpesvirus 5 (ChHV5). In Hawaiian populations, marine leeches (*Ozobranchus* spp.) have been proposed as potential vectors due to high ChHV5 DNA loads (Greenblatt et al., 2004). Other parasites (e.g., blood flukes, barnacles, amphipods) have also been examined, but isopods were not included in that work. Future studies should screen *R. signata* for ChHV5 and evaluate its capacity to acquire and transmit the virus.

## Data Availability

All specimens are deposited in the Crustacea collection from the National Museum, Rio de Janeiro (MNRJ 31969-31975) and data is provided within the manuscript.
